# Analysis of the antimicrobial and anti-caries effects of TiF_4_ varnish under microcosm biofilm formed on enamel

**DOI:** 10.1590/1678-7757-2017-0304

**Published:** 2018-02-15

**Authors:** Beatriz Martines de Souza, Constantino Fernandes, Priscila Maria Aranda Salomão, Layla Reginna Silva Munhoz de Vasconcelos, Flaviana Bombarda de Andrade, Ana Carolina Magalhães

**Affiliations:** 1 Universidade de São Paulo Universidade de São Paulo Faculdade de Odontologia de Bauru Departamento de Ciências Biológicas BauruSão Paulo Brasil Universidade de São Paulo, Faculdade de Odontologia de Bauru, Departamento de Ciências Biológicas, Bauru, São Paulo, Brasil.; 2 Universidade de São Paulo Universidade de São Paulo Faculdade de Odontologia de Bauru Departamento de Dentística, Endodontia e Materiais Odontológicos BauruSão Paulo Brasil Universidade de São Paulo, Faculdade de Odontologia de Bauru, Departamento de Dentística, Endodontia e Materiais Odontológicos, Bauru, São Paulo, Brasil.

**Keywords:** Dental biofilm, Enamel caries, Fluoride, Titanium, Varnish

## Abstract

Titanium tetrafluoride (TiF_4_) is known for interacting with enamel reducing demineralization. However, no information is available about its potential antimicrobial effect.

**Objectives:**

This study evaluated the antimicrobial and anti-caries potential of TiF_4_ varnish compared to NaF varnish, chlorhexidine gel (positive control), placebo varnish and untreated (negative controls) using a dental microcosm biofilm model.

**Material and Methods:**

A microcosm biofilm was produced on bovine enamel previously treated with the varnishes, using inoculum from human saliva mixed with McBain saliva, under 0.2% sucrose exposure, for 14 days. All experiments were performed in biological triplicate (n=4/group in each experiment). Factors evaluated were: bacterial viability (% dead and live bacteria); CFU counting (log_10_ CFU/mL); and enamel demineralization (transverse microradiography – TMR). Data were analysed using ANOVA/Tukey's test or Kruskal-Wallis/Dunn's test (p<0.05).

**Results:**

Only chlorhexidine significantly increased the number of dead bacteria (68.8±13.1% dead bacteria) compared to untreated control (48.9±16.1% dead bacteria). No treatment reduced the CFU counting (total microorganism and total *streptococci*) compared to the negative controls. Only TiF_4_ was able to reduce enamel demineralization (ΔZ 1110.7±803.2 vol% μm) compared to both negative controls (untreated: ΔZ 4455.3±1176.4 vol% μm).

**Conclusions:**

TiF_4_ varnish has no relevant antimicrobial effect. Nevertheless, TiF_4_ varnish was effective in reducing enamel demineralization under this model.

## Introduction

Dental biofilm is defined as a community of microorganisms that colonizes the oral cavity, dimensionally arranged and enclosed into an extracellular matrix rich in polysaccharides, proteins/amino acids, environmental DNA (eDNA) and minerals. The exposure to sucrose from diet may favor the development of a cariogenic biofilm rich in acidogenic and aciduric bacteria and extracellular polysaccharides^25^. Hygiene habits, diet, salivary flow and antimicrobial agents may modulate the quantity and quality of the dental biofilm^19^. Considering the protective factors, fluorides and antimicrobials are among the most studied agents^5^^,^^6^^,^^12^^,^^14^^,^^21^^–^^23^.

Fluoride has anticaries effect mainly due to its action reducing demineralization and improving remineralization of the tooth structure. Secondarily, it can provide some antimicrobial effect by reducing bacterial metabolism and interfering in protons extrusion^27^. Studies have shown that certain concentrations of fluoride (10, 50 and 125 ppm F^-^, 5 min/day) are effective in reducing acid production and acid tolerance as well as extracellular polysaccharide formation of *Streptococcus mutans (S. mutans)* biofilm^22^. Recently, Pandit et al.^20^ (2015) showed that 1 min of application of ≥300 ppm F was able to control cariogenic biofilm through inhibition of virulence properties. All previous studies have been done using a monospecies biofilm (46-h to 74-h-old biofilm) and testing NaF as F^-^ source^20^^–^^22^. Generally, NaF affects the virulence factors, but not the bacteria viability^20^.

The antimicrobial effect of fluoride depends on its concentration^20^^,^^21^. Varnish is the highest fluoride concentrated vehicle, with the advantage of having resinous base, which allows a long contact time with the tooth surface^16^. Most varnishes contain NaF as active agent, which has shown to be able to protect the teeth against dental caries when applied twice a year (46% of preventive fraction in permanent dentition)^16^.

On the other hand, our research group has tested the anticariogenic effect of an experimental 4% TiF_4_ varnish compared to 5.42% NaF varnish under abiotic environment^15^. Our results have shown greater effect of TiF_4_ varnish compared to NaF varnish due to its chemical reaction with enamel surface, promoting deposition of Ti compounds with high acid resistance. However, none of our studies have tested its potential as antimicrobial agent, another possible mechanism of action related to fluorides. We expected that the glaze layer produced by TiF_4_ varnish could alter the microorganism adhesion and, consequently, the biofilm growth and viability.

The use of microcosm biofilm, produced from microorganisms in human saliva, can benefit studies with monospecies or multispecies biofilms, allowing the presence of high number of microorganisms and interactions between them in the presence of fluoride or antimicrobial agents. Considering that 1) most studies on antimicrobial action of fluoride have been done using monospecies or dual-species^4^^,^^12^^,^^20^^–^^22^ and 2) the lack of knowledge on the antimicrobial effect of TiF_4_, the aim of this study was to compare the antimicrobial and anticariogenic effects of TiF_4_ varnish with NaF varnish, chlorhexidine gel (positive control), placebo varnish (without any active agent) and untreated specimens (negative controls) using a microcosm biofilm model on bovine enamel.

This research tested the following null hypotheses: 1) There is no significant difference between the fluoride varnishes and positive control on the microbial viability; 2) There is no significant difference between the fluoride varnishes and positive control on CFU counting for total microorganisms and total *streptococci*; 3) There is no significant difference between the fluoride varnishes and positive control in reducing enamel demineralization.

## Material and methods

### Saliva collection

This study was firstly approved by the local Ethical Committee (CEEA 38143714.7.0000.5417). Saliva was collected from 2 healthy donors, who fit the following inclusion criteria: 1) normal salivary flow (stimulated saliva flow >1 mL/min and non-stimulated saliva flow >0.3 mL/min), 2) with previous history of caries, but no current active caries (no active white spot and/or cavitated lesions), 3) with no gingivitis (red or blooding gingival tissue) and 4) with no history of antibiotic intake in the last 3 months. Prior to the day of collection, the donors did not brush their teeth. Furthermore, they were not allowed to ingest food or drinks in the last 2h before saliva collection. The saliva was collected under stimulation by chewing a rubber material for 10 min during the morning. After collection, saliva was diluted in glycerol (70% saliva and 30% glycerol). Aliquots of 1 mL were stored in -80*°C*^23^.

### Tooth sample preparation and treatment

One hundred twenty (60 for viability assay and 60 for CFU counting) enamel samples (4 mm x 4 mm) were prepared from bovine teeth, using a semi-precision cutting machine (Buehler; Lake Bluff, Illinois, USA). The samples were fixed in acrylic discs with wax and polished in a metallographic polishing machine (Arotec; Cotia, São Paulo, Brazil) using water-cooled silicon-carbide discs (600-grade papers ANSI grit. Buehler, USA) to achieve a flat surface and to standardize the surface roughness of approximately 0.133±0.029 μm. The average surface roughness (Ra) was assessed using a contact profilometer and Mahr Surf XCR 20 software (Mahr; Göttingen, Lower Saxony, Germany). Two thirds of the enamel surfaces were protected with nail varnish to obtain control areas for the transverse microradiography (TMR) analysis.

Enamel samples were randomly divided among study groups according to the Ra values: A) 4.0% TiF_4_ varnish (pH 1.0, 2.45% F^-^); B) 5.42% NaF varnish (pH 5.0, 2.45% F^-^); C) 2% chlorhexidine gel – CHX (pH 6.0) – *Positive control*; D) placebo varnish (pH 5.0) and E) untreated specimens – *Negative controls*. The varnishes were produced by FGM Produtos Odontológicos LTDA (Joinville, Santa Catarina, Brazil) and contained artificial resin as base and ethanol as solvent. During 6 h-treatment^7^^,^^15^, the samples were immersed in remineralizing solution^13^. Thereafter, the varnishes and gel were removed using scalpel blade and the samples were cleaned with swab soaked in acetone-water solution (1:1). Two-thirds of the samples surfaces were protected again and they were then stored in artificial saliva overnight, until they were used for the microcosm biofilm formation.

### Microcosm biofilm formation

The human saliva was defrosted and mixed with McBain artificial saliva^18^ in a proportion of 1:50. The McBain saliva contained 2.5 g/L mucin from porcine stomach (type II), 2.0 g/L bacteriological peptone, 2.0 g/L tryptone, 1.0 g/L yeast extract, 0.35 g/L NaCl, 0.2 g/L KCl, 0.2 g/L CaCl_2_, 0.1 g/L cysteine hydrochloride, 0.001 g/L hemin, 0.0002 g/L vitamin K1, at pH 7.0. All reagents were from Sigma-Aldrich (St. Louis, Missouri, USA). The solution of human saliva and McBain saliva was added to each well containing a treated enamel sample (v=1.5 mL/well) in a 24-well plate, which was incubated at 5% CO_2_ and 37°C, for 8 h. Subsequently, the enamel samples were transferred to wells containing fresh McBain saliva with 0.2% sucrose and incubated at the same conditions. After 16 h, the samples were again transferred to new wells containing fresh McBain saliva with 0.2% sucrose and incubated for 24 h at the same conditions^30^. This procedure was repeated each 24 h, for a total time of 14 days.

### Bacterial viability analysis

After 14 days, the samples were immersed in phosphate-buffered saline (PBS) solution (twice for 5 s) under stirring to remove unattached bacteria. The biofilm was stained using the nucleic acid markers diluted in PBS (1 mL PBS + 1 μL SYTO9 + 1 μL propidium iodide, 10 μL/well) (Kit Live & Dead^®^ cells viability assay, Thermo Fisher Scientific; Waltham, Massachusetts, USA) for 15 min in a dark environment. Live bacteria were stained with SYTO9 producing a green fluorescence and dead lysed bacteria were stained with propidium iodide/SYTO9 producing a red fluorescence^10^. Biofilm was examined using confocal laser scanning microscope (Leica TCS SPE, Leica Mannheim; Wetzlar, Hesse, Germany) and Leica Application Suite-Advanced Fluorescence software (LAS AF, Leica Mannheim; Wetzlar, Hesse, Germany). Three images (275 μm^2^) were captured from each sample surface and analysed using BioImage L 2.0 software, to quantify the live and dead bacteria (%).

### Colony forming unit (CFU) counting

The samples were immersed in PBS solution (twice for 5 s) under stirring to remove unattached bacteria. The samples were then transferred to microtubes containing 1 mL of Brain Heart Infusion (BHI, Difco; Lawrence, Kansas, USA) and vortexed at 2400 rpm for 30 s (vortex 251, Fanem; Guarulhos, São Paulo, Brazil). The bacterial suspension was then diluted to 10^-4^ and spread on petri dishes (50 μL/dish) containing two different types of agar for CFU counting: 1) tryptic soy blood agar with 5% sheep blood for total microorganisms^5^ and 2) mitis salivarius agar (MSA) containing 15% sucrose and 1% potassium tellurite for total *streptococci*^14^. The dishes were stored at 5% CO_2_ and 37°C. After 72 h, the CFU numbers were counted and transformed in log_10_ CFU/mL.

### Transverse microradiography (TMR)

After cleaning, the enamel samples were sectioned at the center of the surface, perpendicularly to the orientation of the protective nail varnish, allowing all enamel areas (sound and demineralized) to be included in the TMR specimens. The specimens were polished to obtain slices with 80-100 μm of thickness. Enamel slices were fixed in a sample-holder together with an aluminium calibration step wedge with 14 steps. A microradiograph was taken using an x-ray generator (Softex; Tokyo, Japan) on the glass plate at 20 kV and 20 mA (at a distance of 42 cm) for 13 min. The glass plates were developed for 7 min, rinsed in deionized water, fixed for 7 min in a dark environment, and then rinsed in running water for 10 min and air-dried (all procedures were done at 20°C). The developed plate was analysed using a transmitted light microscope fitted with a 20x objective (Zeiss, Oberkochen; Baden-Württemberg, Germany), a CCD camera (Canon; Tokyo, Japan), and a computer. Two images *per* sample were taken using data-acquisition (version 2012) and interpreted using calculation (version 2006) software from Inspektor Research System bv (Amsterdam, North Holland, The Netherlands). The mineral content was calculated considering the density of the mineral to be 3.15 kg L^-1^ and 87 vol% of mineral content for the sound enamel. The lesion depth (LD, μm), the integrated mineral loss (ΔZ, vol% μm) and the average mineral loss over the lesion depth (R, vol%) were calculated^7^.

### Statistical number and analysis

All biofilm analyses were done in biological triplicate (n=4/each experiment, final number=12) while all enamel samples from both analyses (% dead and log_10_ CFU/mL) were applied for TMR (final number=24, ΔZ, LD and R). The sample number calculation for biofilm analysis was based on previous work^30^. Data were statistically analysed using the software Graph Pad Instat for Windows (GraphPad Software; La Jolla, California, USA). Normal distribution and homogeneity were checked using Kolmogorov-Smirnov and Bartlett's tests, respectively. Ordinary ANOVA followed by Tukey's test were applied to compare the different treatments for all analyses except LD. In case of LD, Kruskal-Wallis followed by Dunn's test were performed. A significance level of 5% was considered for all statistical tests.

## Results

**Figure 1:**
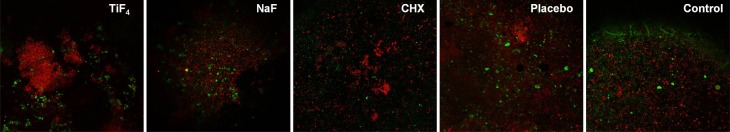
Representative confocal images of the biofilm formed on enamel samples. *CHX=chlorhexidine

**Figure 2:**
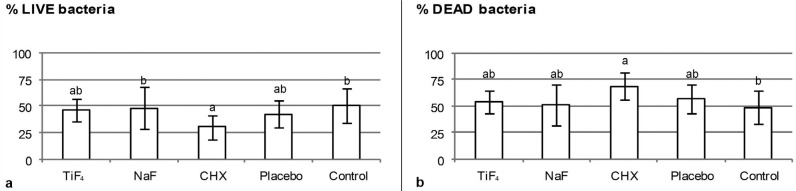
a) Percentage of live bacteria and b) Percentage of dead bacteria in biofilm formed on enamel after treatments. Different letters show significant differences among treatments. ANOVA and Tukey's test (p<0.02). *CHX=chlorhexidine

In respect to the biofilm viability, only chlorhexidine was able to significantly increase the number of dead bacteria compared to untreated control (p<0.002), but it did not significantly differ from placebo. No significant differences were found between fluoride treatments or among fluoride treatments and negative or positive control. Figure 1 shows a representative confocal picture of the biofilm from each treatment group and Figure 2 shows the viability data. CFU counting for total microorganism and total *streptococci* showed no significant differences among treatments (p>0.05) (Table 1).

**Table 1:** Mean and standard deviation (SD) of colony forming units (CFU) counting (log_10_ CFU/mL) for total microorganisms and total *streptococci* in the biofilm formed on enamel after applying the treatments tested

Treatment	Total microorganism	Total *Streptococci*
TiF_4_	7.04±0.20^a^	6.83±0.26^a^
NaF	7.02±0.18^a^	6.97±0.22^a^
Chlorhexidine	6.97±0.30^a^	6.92±0.25^a^
Placebo	7.14±0.22^a^	6.93±0.25^a^
Control	7.03±0.09^a^	7.04±0.17^a^

Similar letters show no significant differences among the treatments (*per* column). ANOVA and Tukey's test (n=12, p>0.05)

**Figure 3:**
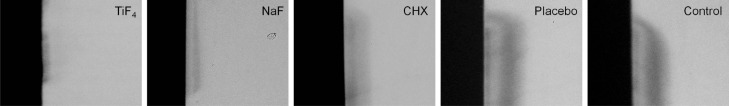
Representative transverse microradiography (TMR) pictures (20x) of artificial enamel lesions created using microcosm biofilm after applying the treatments tested. The largest lesions are seen for placebo varnish and untreated groups; while the smallest lesion is seen for TiF_4_ followed by NaF varnish groups. The CHX-treated enamel had an intermediate lesion size. The % of lesions with a surface layer was: 40% TiF_4_, 61.5% NaF, 68.8% CHX, 50% placebo varnish and 73% control. *CHX=chlorhexidine

TiF_4_ and NaF varnishes were similarly able to significantly reduce the integrated mineral loss and the average mineral loss compared to the untreated group and chlorhexidine, while only TiF_4_ was significantly different from placebo varnish. Both TiF_4_ and NaF reduced lesion depth compared to control, but only TiF_4_ was significantly different from placebo. Treatment with chlorhexidine did not reduce enamel demineralization (Table 2). Figure 3 shows a representative TMR picture of demineralized enamel from each treatment group.

**Table 2:** Integrated mineral loss (ΔZ, vol% μm), average mineral loss (R, vol%) and lesion depth (LD, ?m) of enamel from each treatment group

Treatment	ΔZ (%vol. μm)	R (%vol)	LD (μm)
TiF_4_	1100.7±803.2^c^	19.9±3.7^c^	68.3±33.4^c^
NaF	1680.0±538.3^bc^	20.9±4.9^bc^	86.9±29.1^bc^
Chlorhexidine	3262.7±909.6^a^	33.9±6.4^a^	104.3±29.2^abc^
Placebo	3374.6±1636.9^ab^	28.9±6.5^ab^	162.3±64.7^ab^
Control	4455.3±1176.4^a^	29.4±5.3^a^	156.1±27.1^a^

Different letters show significant differences among the treatments (*per* column). ΔZ and R-values are displayed as mean ± SD (ANOVA and Tukey's test, p<0.0001). LD is presented as median ± CI (Kruskal-Wallis and Dunn's test p<0.0001). From 24 samples, the final number was: TiF_4_ (n=14), NaF (n=12), CHX (n=15), placebo (n=11) and control (n=15). The samples were lost during the preparation for TMR.

## Discussion

This experimental model allowed the formation of a biofilm based on saliva, reproducing the complex relationship between salivary components and bacterial species, as proposed in the microcosm biofilm model. A sucrose supplementation was provided to favor the proliferation of cariogenic species in the biofilm, which can induce tooth demineralization^28^.

Microcosm model is a validated method to test the effects of complex biofilms on tooth^24^. The donors were submitted to a complete screening for a better selection of the source of microorganisms (saliva). However, a recent study investigated the effect of different types of inoculum (saliva and dental biofilm) from caries-active and caries-free individuals on the cariogenic potential of biofilm produced *in vitro*. The authors found that the cariogenic potential of the biofilms, produced under identical conditions *in vitro*, is similar regardless of the microorganism's source^25^. Therefore, the criteria applied for subjects' selection may be negligible for this model.

The metabolic activity of the microorganisms, a determinant of the development of the disease, is influenced by the conditions (the atmospheric condition as well as the type of nutrient) of the environment during the biofilm formation^26^. In our study, the microcosm biofilm was created as previously described^30^ and grown under sucrose exposure, at 5% CO_2_ and 37°C for 14 days, allowing the formation of a thicker biofilm and the production of an artificial caries lesion with LD of 150 μm. As positive control, we applied a commercial chlorhexidine gel that was able to reduce the microorganism viability in our study. However, chlorhexidine had no effect on CFU counting.

We believe that chlorhexidine affects the viability of microorganisms not directly involved with dental caries, which are in lower quantity in our microcosm biofilm and, therefore, it did not have significant influence on the total microorganisms CFU counting. Other possible explanation is that the bacteria affected by chlorhexidine in the biofilm recovered its viability under favorable conditions provided during the cultivation (fresh medium with nutrients) for the CFU counting. The result suggests that chlorhexidine had no residual effect on the bacteria after their growth in a specific medium for 72 h. If chlorhexidine had been applied daily as done by other authors^17^, its residual effect could have been seen. However, it induces some side effects as tooth discoloration and astringent taste under uninterrupted use^2^. Therefore, considering the side effects and allowing comparison with the varnishes, we applied chlorhexidine once at the beginning of the experiment.

We already know that TiF_4_ can reduce enamel demineralization^6^^,^^7^^,^^15^ mainly due to its reaction with hydroxyapatite producing an acid resistant layer. This layer is composed of hydrated titanium phosphate, titanium oxide and calcium fluoride and behave significantly better (more acid resistant) in protecting enamel than CaF_2_^-^, such as the layer produced by NaF^6^. We expected that this layer would interfere in the microorganism adhesion and biofilm growth and activity; but we found no antimicrobial effect of TiF_4_ varnish under this model. Recently, Eskandarian, et al.^9^ (2017) showed antimicrobial effect of TiF_4_, however, using a model and a fluoride preparation that are far to simulate the real conditions. They applied a planktonic model (broth dilution and disk diffusion) and neutralized TiF_4_ solution to achieve a pH of 7.2, which is shown to negatively impact its effect on the tooth^29^. Furthermore, the minimum inhibitory concentration (MIC) and minimum bactericidal concentration (MBC) values for *S. mutans* were extremely high for TiF_4_ (12.5% and 25%, respectively, inapplicable in the oral cavity), and not significantly different from NaF.

Regarding the antimicrobial effect of fluorides, the literature is restricted to NaF. The understanding about the antimicrobial effect of NaF is mainly based on studies using a short-term *S. mutans* biofilms^20^^–^^22^. Generally, the aforementioned studies have shown that NaF can reduce acid production and tolerance of *S. mutans*, a dose-dependent effect (mainly in a range of 10 and 100 ppm F^-^). Biomass and viability are only affected when NaF is often applied (two times a day) and extracellular polysaccharide production is only disturbed when high F^-^ concentrations are tested (>300 ppm F^-^). Recently, Dang, et al.^8^ (2016) showed that a short fluoride treatment (1-8 min, representing an exposure to fluoride mouthrinse or toothpaste) does not sustain anti-acidogenic activity of NaF (0-2000 ppm F^-^) against *S. mutans* biofilm, since the acid production recovers with time. They also showed that the bacteria viability is not affected by fluoride, in agreement with our results.

Additionally, Jung, et al.^12^ (2016) demonstrated that NaF (0-100 ppm F^-^) reduced the proportion and bio-volume of *S. mutans* biofilm*,* but did not decrease those of *S. oralis* biofilm under a short-term and dual-species biofilm model; a result that was attributed to the inhibitory effect of fluoride on extracellular polysaccharide production. There is no study using a complex biofilm model, as microcosm biofilm, to test the antimicrobial effect of fluorides so far. There is only one *in situ* study that tested the effect of AmF/NaF mouthrinse on the adhesion of bacteria to enamel and dentin, which showed some inhibition only for dentin, but not for enamel^10^, in agreement with our study.

Our study is the first one dealing with microcosm biofilm to test the antimicrobial effect of NaF and TiF_4_ varnishes. It is important to highlight that all aforementioned studies only analyzed biofilm and not the tooth alterations, because almost all works produced biofilm on hydroxyapatite discs. Therefore, our study brings further important information as it combined the biofilm analysis with the enamel demineralization quantification.

In opposition to the study by Chau, et al.^4^ (2014), we removed the fluoride varnish before the biofilm formation to better simulate the oral environment, since varnishes do not permanently stay on the tooth. We wanted to check if the layer produced by NaF (rich in CaF_2_) or TiF_4_ (rich in titanium phosphate, titanium oxide and CaF_2_)^6^ varnish would, in turn, have any influence on biofilm growth and viability.

Further studies should be conducted to identify the potential species of *streptococci* or other bacteria in microcosm biofilm that could be affected by TiF_4_. Bowden and Hamilton^3^ (1989) have already discussed the existing competition between *S. mutans* and *Lactobacillus casei* under conditions of varying environmental pH and in the presence of fluoride*.* In addition to these traditionally known species, recent studies pointed out the presence of other microbial species such as *Scardovia wiggsiae* and *Bifidobacterium spp*., which are acid-resistant and associated with dental caries^11^.

Our results allowed us to accept all null hypotheses except the last one. Both NaF and TiF_4_ varnishes were unable to reduce the bacteria viability in agreement with the works of Pandit, et al.^20^^-^^22^ (2015, 2013, 2011). The lack of antimicrobial effect of the fluoride varnishes may be explained by the consumption of the fluoride-rich layer on enamel over time in a long-term biofilm model, as applied in this study. We speculate that the antimicrobial effect of fluoride varnishes could have been detected if a short-term (46-74 h) *S. mutans* biofilm had been applied. However, short-term biofilm usually does not allow a real formation of a caries lesion. On the other hand, *S. mutans* biofilm does not simulate the complexity of the *in vivo* biofilm. Different results could also have been obtained with daily application of a TiF_4_ mouthrinse instead of a unique application of TiF_4_ varnish, but this comparison was not the aim of the study.

Despite being antimicrobial, chlorhexidine did not reduce enamel demineralization (anticariogenic effect) under this model, which is supported by previous clinical trials showing no benefits of the application of chlorhexidine varnish in the prevention of caries in children and adolescents^1^. On the other hand, both fluoride varnishes were able to reduce enamel demineralization, with TiF_4_ being more effective as it significantly differed from placebo varnish, in agreement with the results found in our previous abiotic study^15^. The microcosm biofilm model is much more aggressive than the abiotic model and, even under high cariogenic model, TiF_4_ was still more effective against enamel demineralization. A recent *in situ* study^7^ provides more support for the benefit of applying TiF_4_ varnish instead of NaF varnish. TiF_4_ varnish was the only treatment able to improve enamel remineralization regardless of the cariogenic activity, while NaF varnish failed in preventing further demineralization under high cariogenic activity (biofilm under 20% sucrose 8 times a day) *in situ*.

## Conclusions

TiF_4_ varnish has no relevant antimicrobial effect. Nevertheless, TiF_4_ varnish was effective in reducing enamel demineralization (anticariogenic effect) under this model.
